# Validating a measure for eco-anxiety in Portuguese young adults and exploring its associations with environmental action

**DOI:** 10.1186/s12889-023-16816-z

**Published:** 2023-10-02

**Authors:** Francisco Sampaio, Tiago Costa, Luísa Teixeira-Santos, Lara Guedes de Pinho, Carlos Sequeira, Sílvia Luís, Ana Loureiro, Jerônimo C. Soro, Juan Roldán Merino, Antonio Moreno Poyato, Juan Segundo Peña Loray, Andrea Rodríguez Quiroga, Léan V. O’Brien, Teaghan L. Hogg, Samantha K. Stanley

**Affiliations:** 1 Nursing School of Porto, Porto, 4200-072 Portugal; 2 CINTESIS@RISE, Nursing School of Porto (ESEP), Porto, 4200-450 Portugal; 3 Centro Hospitalar de Vila Nova de Gaia / Espinho, Vila Nova de Gaia, 4434-502 Portugal; 4 Portuguese Red Cross Northern Health School, Oliveira de Azeméis, 3720-126 Portugal; 5 Nursing School of Coimbra, Coimbra, 3004-011 Portugal; 6 Nursing Department, Universidade de Évora, Évora, 7000-811 Portugal; 7 Comprehensive Health Research Centre, Universidade de Évora, Évora, 7002-554 Portugal; 8 HEI-Lab: Digital Human‐Environment Interaction Labs, Lusófona University, Lisboa, 1749-024 Portugal; 9 Centro de Administração e Políticas Públicas, Instituto Superior de Ciências Sociais e Políticas, Universidade de Lisboa, Lisboa, 1300-663 Portugal; 10 School of Nursing, Campus Docent Sant Joan de Déu – Fundació Privada, Sant Boi de LLobregat, Barcelona, 08830 Spain; 11 Mental Health, Psychosocial and Complex Nursing Care Research Group (NURSEARCH), Universitat de Barcelona, Barcelona, 08007 Spain; 12 Public Health, Mental Health and Maternal-Infant Nursing Department, Nursing College, Universitat de Barcelona, Health Sciences Campus Bellvitge, Barcelona, 08007 Spain; 13 Foundation Turning Point for Health and Sustainability, Barcelona, Spain; 14 Discipline of Psychology, University of Canberra, Canberra, ACT 2617 Australia; 15 School of Medicine and Psychology, Australian National University, Canberra, ACT 2600 Australia

**Keywords:** Climate anxiety, Eco-anxiety, Climate change, Pro-environmental behaviour, Scale validation, Psychometrics

## Abstract

**Background:**

Worsening environmental conditions may amplify people’s emotional responses to an environmental crisis (eco-anxiety). In Portugal, young people seem to be especially concerned about climate change. However, this phenomenon needs to be interpreted using accurate instruments. Thus, this study aimed to validate the Portuguese version of the Hogg Eco-Anxiety Scale (HEAS) in young adults and examine the associations among eco-anxiety, sociodemographic characteristics, and pro-environmental behaviours.

**Methods:**

A survey was administered to 623 Portuguese university students aged between 18 and 25 years. The survey included our Portuguese translation of the HEAS (obtained through a back-translation and pretesting process), a sociodemographic assessment, and questions related to pro-environmental behaviours. Confirmatory factor analysis was conducted to assess the construct validity of the Portuguese version of the HEAS, and global fit indices were used to assess whether the original four-dimensional structure of the scale was reproduced. The reliability of the Portuguese version of the HEAS was evaluated by Cronbach’s alpha and the intraclass correlation coefficient. Measurement invariance examined sex differences in scale interpretation. Linear regressions were used to detect whether sociodemographic variables predict eco-anxiety and whether eco-anxiety predicts pro-environmental behaviours.

**Results:**

The factorial structure of the original scale was replicated in the Portuguese version of the HEAS, showing good internal consistency, reliability over time and strict invariance between men and women. A higher paternal education level predicted greater eco-anxiety in children. Two dimensions of eco-anxiety—namely, rumination and anxiety about personal impacts on the environment—predicted higher engagement in pro-environmental behaviours.

**Conclusions:**

The translated scale is an appropriate tool to measure eco-anxiety in the Portuguese context and should be used to collect evidence to drive environmental and health policies. An individual’s education level should be considered a determinant of their emotional response to environmental conditions. Importantly, eco-anxiety can act as a protective emotional response to preserving the planet.

**Supplementary Information:**

The online version contains supplementary material available at 10.1186/s12889-023-16816-z.

## Background

Climate change threatens human health [1] and the viability of many ecological systems [2]. We are not on track to prevent the quickly-escalating effects of climate change that have become a major concern worldwide, especially among young people who anticipate tremendous impacts of climate change in their future [3].

The term “eco-anxiety” refers to the anxiety people experience as environmental conditions worsen [4, 5]. Negative effects of eco-anxiety have been identified across different regions, such as Europe, the United States, Canada, the Pacific Islands, Africa, and the Philippines [4]. The media has raised special concerns about eco-anxiety among young people [5], who will inherit a world affected by climate change. Supporting these concerns, there is evidence that younger people experience high levels of anxiety about climate change [3, 6]; as a result, eco-anxiety has caught the attention of researchers, activists, and governmental and nongovernmental organisations (NGOs), who are working to better understand the experience of climate change and its relation with individual well-being and behaviour [6–8].

The Hogg Eco-Anxiety Scale (HEAS) was recently developed to capture four different dimensions of eco-anxiety [7]. It provides a nuanced picture of the ways that people experience eco-anxiety by identifying the extent to which individuals experience affective symptoms (e.g., worrying too much and feeling on edge), behavioural symptoms (e.g., disruptions to one’s sleep, schooling, or socialising), rumination (repeated thoughts about ecological loss), and concern about their impact on the environment. The HEAS has advantages over other measurement tools that capture only feelings of distress [9, 10] and the Climate Anxiety Scale, which measures the cognitive-emotional (e.g., interference with cognition) and functional (e.g., interference with work and study) impairments specifically associated with climate anxiety [6]. The HEAS has been validated in Australia and New Zealand [7, 11] and Turkey [12] and has thus far shown excellent performance [7, 11]. However, an essential next step is to validate the HEAS in other countries, including Portugal. The pattern of eco-anxiety observed internationally is also present in Portugal. Hickman and colleagues [3] found that most young people in Portugal are either extremely or very worried about climate change, and this anxiety impacts most of their daily lives. Like many countries worldwide, Portugal’s climate is becoming warmer and drier due to climate change [13]. Our research facilitates the examination of eco-anxiety in Portugal by developing a translated version for the Portuguese context: the HEAS-PT.

We also contribute to the understanding of who is most at risk of experiencing greater eco-anxiety in Portugal. Limited research suggests that younger age groups experience more climate anxiety [6], and women may experience more cognitive-emotional (but not functional) impairment due to climate anxiety than men [14]. Thus, we expected similar differences in eco-anxiety across age demographics (i.e., greater levels of each dimension of eco-anxiety among younger participants) and sex (i.e., higher affective symptoms among women).

We also contribute to the debate about whether eco-anxiety is a potential facilitator or inhibitor of climate action by examining associations between the dimensions of eco-anxiety and pro-environmental behaviour. The psychological literature refers to anxiety as an *activating* emotional state [15], meaning that it is an energising experience that can prompt an individual to act on a threat. There are two conflicting perspectives: that eco-anxiety motivates action [16] and that eco-anxiety leads to eco-*paralysis* and stalls individual behaviour [14, 17]. Sangervo and colleagues [16] found that Finns, who were more anxious about climate change, engaged in more climate action, and that Australians’ eco-anxiety was related to more climate actions (though this relationship was reversed after controlling for climate-related feelings of anger and depression) [18]. However, this research used simple conceptualisations of eco-anxiety to determine how intensely people experience certain feelings. Using a multidimensional conceptualisation capturing the cognitive and functional impairment components of climate anxiety, Clayton and Karazsia [6] found no association with behaviour among adults in the United States. Within French-speaking populations, Heeren and colleagues [14] showed a positive association between climate anxiety and pro-environmental behaviour that tapered off for those who had higher climate anxiety, suggesting diminishing returns of climate anxiety on behaviour among individuals with higher climate anxiety. Current evidence on the behavioural corollaries of climate anxiety is, therefore, limited and conflicting.

The multidimensional HEAS captures a large breadth of information, thus likely revealing distinct associations between its dimensions and pro-environmental behaviours. We expected that the affective and behavioural dimensions would show no unique associations with pro-environmental behaviour based on Clayton and Karazsia’s findings [6]. In contrast, we noted that anxiety about one’s impact on the environment is akin to self-conscious emotions, such as guilt about contributing to the problem. Self-conscious emotions function to regulate behaviour [19], and thus, we expected anxiety regarding personal impact to be associated with reducing one’s impact on the planet by engaging in more pro-environmental behaviours. We also speculated that by keeping environmental impacts front-of-mind, the ruminative element of eco-anxiety would demonstrate a unique positive association with behaviour, although due to the lack of evidence, we made no firm prediction on this point.

## Methods

### Design

This cross-sectional study followed the STROBE guidelines, and data collection was carried out from March 30 to July 7, 2022. In addition, a second survey was provided to the participants 7–10 days after the first survey was completed to assess the test-retest reliability of the HEAS-PT.

### Participants and study setting (sample size)

Portuguese undergraduate students aged 18–25 years were recruited from higher education institutions (HEIs). Each HEI was asked for authorisation to collect data on their undergraduate students. A total of 4 faculties/schools and 12 universities across Portugal agreed to participate in the study. The HEIs were asked to email their students with information about the study and the link to the data collection tool (completed through an online platform). In addition, each HEI was asked to send a gentle reminder to all students one week after they were emailed with the invitation to participate in the study. Participation of the students was voluntary, and no compensation was offered for completing the survey.

The target sample size was based on Comrey and Lee’s recommendations [20], who suggest a graduated scale to determine the sample size for scale development: 100 = poor, 200 = fair, 300 = good, 500 = very good and from 1,000 = excellent. Based on these benchmarks, we decided to include at least 500 undergraduate students.

A total of 623 undergraduate students participated in the study. Participants were, on average, 20.46 years old (SD = 1.83 years), and 81.5% were female. Most participants were single (53.0%) or cohabiting (46.4%). Only six students were members of an environmental association. Most participants (59.7%) grew up in an urban area, and 71.3% live in an urban area. Only 14.3% and 11.2% of the participants reported having a chronic physical disease and a chronic mental disease, respectively.

To analyse the test-retest reliability of the HEAS-PT, we estimated that it had to be fulfilled by at least 196 participants at two time points to detect an intraclass correlation coefficient (ICC) of agreement greater than or equal to 0.60 between the two, considering an alpha value of 0.05 and a power of 90% in a bilateral contrast [21]. A total of 200 participants completed the follow-up survey.

### Measurements

This study measured eco-anxiety by translating the HEAS [7] into Portuguese. The HEAS was originally validated in Australia and New Zealand and comprises four items assessing the affective symptoms of eco-anxiety, three items measuring ruminative thoughts relating to environmental issues, three items measuring impairment in behavioural and social functioning, and three items measuring anxiety about one’s impact on the planet. Thus, the scale comprises 13 items that capture the full experience of eco-anxiety. Responses were measured along a 4-point frequency scale (0 = *not at all*, 3 = *nearly every day*). The original version of the scale presented good psychometric properties, such as test-retest reliability and internal reliability (α > 0.82 for all subscales).

Pro-environmental behaviours were measured using a set of 18 questions adapted from Whitmarsh and O’Neill’s study [22]. A UK review led by DEFRA [23] identified 12 “headline behaviours”, which included low and high environmental impact actions and one-off and regular decisions relating to four behavioural domains: domestic energy/water use, waste behaviour, transport, and eco-friendly shopping. Considering these 12 headline behaviours, Whitmarsh and O’Neill developed a 24-item pro-environmental measure that presented excellent internal reliability (α = 0.82). In this study, we selected 18 of the 24 items proposed by Whitmarsh and O’Neill, with responses measured by a 4-point frequency scale (0 = *never* to 3 = *always*). We decided not to include the six items that relied on home ownership or a high income level (e.g., installing insulation in one’s home, purchasing a low-emission vehicle) and thus were unlikely to apply to our young adult sample.

### Data collection and statistical analyses

The translation and adaptation of the HEAS into European Portuguese followed the recommendations of Boateng and colleagues [24]. Thus, procedures consistent with the International Test Commission guidelines were adopted [25]. Specifically, two independent translations into European Portuguese were performed by two bilingual translators whose native language was European Portuguese. The translations were submitted for appraisal by a committee of experts (an expert in environmental matters, an expert in psychometrics, and two experts in psychiatry and mental health), who analysed the semantic equivalence. Finally, a consensus version of the scale in European Portuguese was obtained. This consensus version was later back-translated into the original language by two bilingual translators whose native language was English. After the back-translation was complete, it was compared with the original version. The panel of experts produced the final version in European Portuguese based on the semantic, idiomatic, and conceptual equivalence assessment. Finally, a pretest of the Portuguese version of the scale was conducted with 20 Portuguese young adults aged between 18 and 25 years (purposive sampling) to assess the time needed to complete each scale and the ease of understanding the items. After this process was completed, it was concluded that no further changes to the items were necessary (see Additional File 1).

To validate the HEAS for the Portuguese population and to explore the associations of eco-anxiety with, for instance, pro-environmental behaviours, a data collection tool was used to assess demographic variables such as age, sex, marital status, years of education, association membership status (e.g., environmental association), the type of area in which the participant grew up (urban vs. rural), the current residence area (urban vs. rural), paternal and maternal educational attainment, chronic physical illness status, and chronic mental illness status. The data collection tool also included the HEAS (the scale we intended to validate for the Portuguese population) and a set of questions addressing pro-environmental behaviours.

Data were analysed using IBM SPSS for Windows, version 27 [26]. The construct validity of the HEAS was determined by confirmatory factor analysis (CFA). The model was estimated using Eq. 6.3 for Windows [27]. CFA is a suitable choice to analyse the internal structure of a scale when there is a well-established theoretical basis and/or existing studies that have already explored or confirmed its factor structure, which has already been done by Hogg et al. [7]. The maximum likelihood method was used to determine if the original four-dimensional structure of the scale was reproduced in the HEAS-PT, which was suggested to be the best fitting model to measure the multidimensional construct of eco-anxiety, according to Hogg et al. [7]. Therefore, no other models were tested. In this study, the following global fit indices were considered: the goodness-of-fit index (GFI), adjusted goodness-of-fit index (AGFI), comparative fit index (CFI), Bentler–Bonett normed fit index (BBNFI), Bentler–Bonett nonnormed fit index (BBNNFI), standardised root mean square residual (SRMR) and root mean square error of approximation (RMSEA). The criterion for defining a good overall fit was set at > 0.90 (good fit) or > 0.95 (excellent fit) for the GFI, AGFI, CFI, BBNFI, and BBNNFI and < 0.08 (good fit) or < 0.005 (excellent fit) for the SRMR and RMSEA [20, 28]. Reliability was analysed through internal consistency with Cronbach’s alpha, considering an alpha of 0.70 as the minimum acceptable value [29]. The homogeneity coefficient of the corrected items was also calculated by estimating the correlations of each item with the total scale. A correlation of 0.30 was accepted as the lower limit [30]. Test-retest reliability was calculated using the ICC (based on a single-rating, absolute agreement, two-way mixed effects model) for each factor. The ICC values ​​varied between 0 and 1. Test-retest reliability is considered very good for values ​​>0.90, good for values ​​from 0.71 to 0.90, moderate for values ​​from 0.51 to 0.70, mediocre for values ​​from 0.31 to 0.50, and poor or null for values under 0.31 [29].

Measurement invariance was tested by multigroup confirmatory factor analyses to cross-validate the established HEAS-PT factor across sexes (male and female). This procedure allowed for the examination of whether respondents of different sexes interpreted the same measure in a conceptually similar way. Determination of the measurement invariance was accomplished by a multistep process [31, 32]: the calculation of an unrestricted model, the calculation of a measurement weight model, the calculation of a measurement intercept model and the calculation of a measurement residuals model. For a χ^2^/df value ≤ 3, absolute RMSEA and SRMR values ≤ 0.06 and CFI values above 0.95 were considered acceptable [33]. Changes in χ^2^ were observed when comparing models. Additionally, a comparison was made among the RMSEA, SRMR, and CFI values.

To determine whether eco-anxiety dimensions were predicted by sociodemographic variables (sex, age, education level, living area and parental education attainment), multiple linear regressions were carried out. The same analysis was used to test whether eco-anxiety dimensions predicted pro-environmental behaviours.

This study followed the Declaration of Helsinki [34]. Ethical approval was obtained from the Ethical Committee of the University Fernando Pessoa (ESS/PI – 269/22). All respondents provided informed consent.

## Results

### Psychometric properties of the HEAS-PT

Confirmatory factor analysis was used to test the hypothesised factor structure of the HEAS-PT. The significant chi-square (χ^2^) obtained could indicate that the model did not fit the data. However, this statistic is typically influenced by several factors and cannot be used as a sole indicator for model‐data fit [35]. For this reason, the model fit was assessed based on multiple fit indices (Table 1). Most indices yielded a good to excellent model fit.

**Table 1:** Indices of the goodness of fit of the confirmatory model

Index	Model value	Interpretation
BBNFI	0.961	Excellent fit
BBNNFI	0.961	Excellent fit
CFI	0.971	Excellent fit
GFI	0.941	Good fit
AGFI	0.921	Good fit
SRMR	0.047	Excellent fit
RMSEA	0.057	Good fit

BBNFI: Bentler–Bonett Normed Fit Index. BBNNFI: Bentler–Bonett Non-Normed Fit Index. CFI: Comparative Fit Index. GFI: Goodness-of-Fit Index. AGFI: Adjusted Goodness-of-Fit Index. SRMR: Standardised root mean squared residual. RMSEA: Root mean standard error of approximation.

**Fig. 1:**
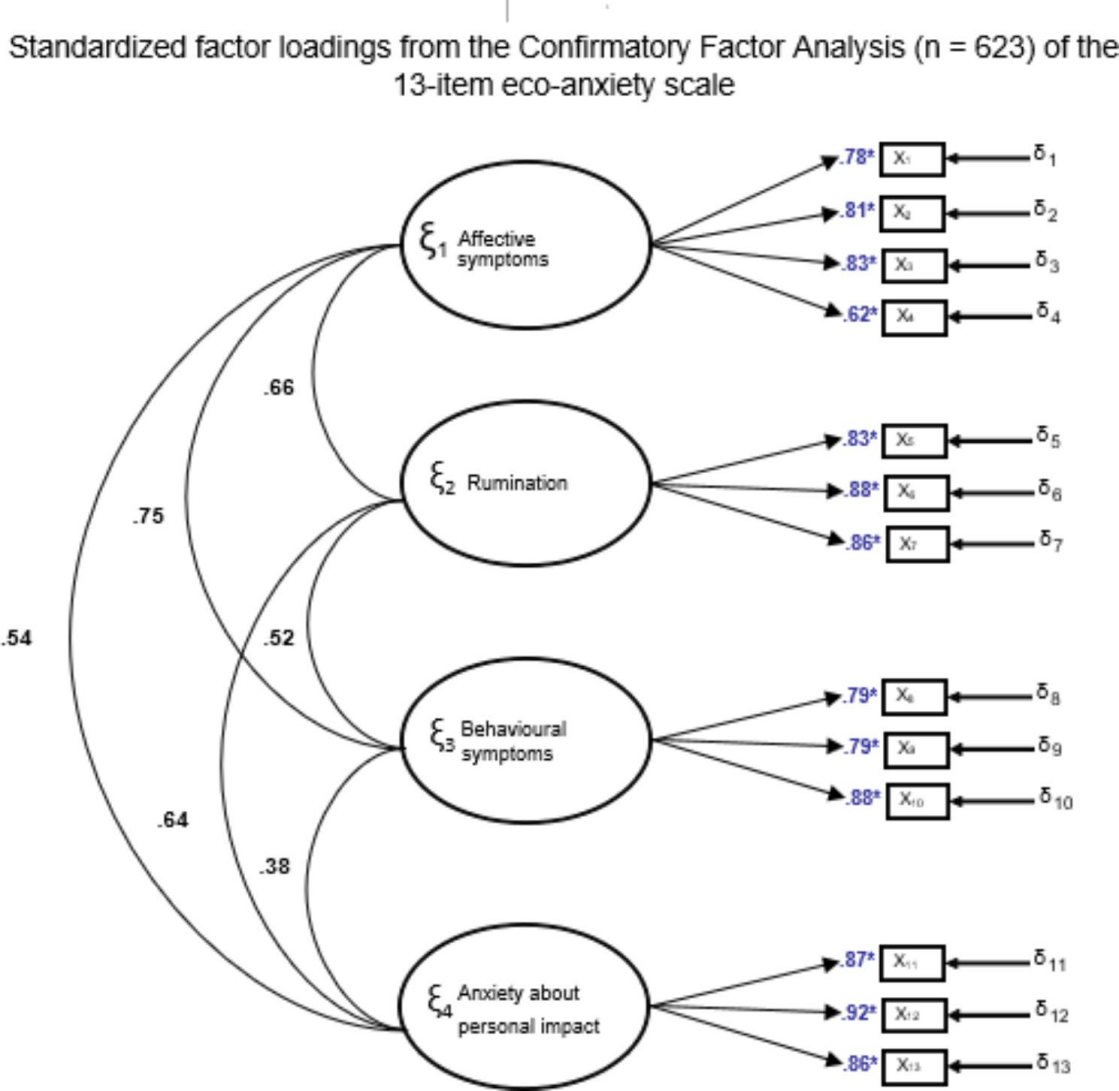
Graphical structure and standardised factor loadings for the HEAS-PT

A graphical representation of the tested model is presented in Fig. 1. The lowest factor loading was 0.62, suggesting that each item was strongly associated with the respective dimension.

Table 2 shows the high internal consistency of the HEAS-PT subscales, ranging from 0.85 to 0.92. Mean values were below 1, suggesting a low average frequency of eco-anxiety symptoms.

**Table 2:** Indices of reliability (Cronbach’s alpha) and central tendency values

Subscale	α without item	Mean	Standard Deviation
1. Affective symptoms: α = 0.847			
Item 1	0.787	0.79	0.782
Item 2	0.794	0.49	0.730
Item 3	0.783	0.73	0.895
Item 4	0.852	0.85	0.789
2. Rumination: α = 0.893			
Item 5	0.866	0.79	0.782
Item 6	0.829	0.49	0.730
Item 7	0.848	0.55	0.744
3. Behavioural symptoms: α = 0.861			
Item 8	0.821	0.57	0.855
Item 9	0.832	0.42	0.733
Item 10	0.759	0.60	0.817
4. Anxiety about personal impact: α = 0.916			
Item 11	0.891	0.88	0.823
Item 12	0.851	0.85	0.818
Item 13	0.896	0.92	0.849

α: Cronbach’s alpha

The HEAS-PT scores from Time 1 and Time 2 (7–10 days later) were correlated to evaluate stability over time. The intraclass correlation coefficients (ICCs) were computed to establish the test-retest reliability of the scale (Table 3). The ICC of all subscales ranged between 0.62 and 0.75, demonstrating moderate to good reliability over time.

**Table 3:** Intraclass correlation coefficient test-retest (n = 200)

Subscale	ICC	CI 95%
1. Affective symptoms	0.749	(0.680–0.805)
2. Rumination	0.629	(0.538–0.706)
3. Behavioural symptoms	0.621	(0.528–0.699)
4. Anxiety about personal impact	0.750	(0.682–0.805)
		–

ICC: Intraclass correlation coefficient (based on a single-rating, absolute agreement, two-way mixed effects model). CI: Confidence interval

### Measurement invariance testing

Measurement invariance was tested to verify that the structural characteristics of the HEAS-PT did not vary between men and women [36]. Following Byrne’s recommendations [31], measurement invariance was examined using a multistep process. First, a configural invariance model was tested using the same factorial structure across sexes with no parameter restrictions (M1: Unrestricted Model). Second, a metric invariance model was tested, constraining factor loadings to be invariant between men and women (M2: Measurements Weight Model). Third, a strong invariance model was estimated, in which both factor loadings and item intercepts were constrained to be invariant between men and women (M3: Measurement Intercepts Model). Finally, a strict invariance model was run in which factor loadings, item intercepts and residual variances were invariant between men and women (M4: Measurement Residuals Model). An estimator robust to maximum likelihood was used for all models.

These models followed Mueller and Hancock’s guidelines [32] for configural invariance: For a χ^2^/df value ≤ 3, absolute RMSEA and SRMR values ≤ 0.06 and CFI values above 0.95 [33] were considered acceptable. Changes in χ^2^ were observed to compare configural, metric, strong, and strict invariance. Because these indicators may be affected by sample size, especially when comparing unbalanced groups, changes in the CFI value ≤ 0.005, RMSEA value ≤ 0.010, and SRMR value ≤ 0.025 were considered consistent with a hypothesis of invariance [37].

The results for configural invariance (M1) showed a good fit (χ^2^/df (2.560), CFI = 0.967, RMSEA = 0.050 and SRMR = 0.040). The same results were shown for metric invariance (M2), indicating a good fit (CFI = 0.963, RMSEA = 0.051 and SRMR = 0.050). Comparing M1 with M2, similar values were found, considering the minimum changes accepted in the CFI, RMSEA, and SRMR. The results for strong invariance (M3) showed a good fit, very similar to those for metric invariance (CFI = 0.962, RMSEA = 0.049 and SRMR = 0.051). A comparison between M2 and M3 showed minimal differences. Additionally, the results for strict invariance (M4) showed good fit (CFI = 0.960, RMSEA = 0.044 and SRMR = 0.083), with minimal differences between M3 and M4 (Tables 4 and 5). These results suggested invariance of the HEAS-PT across sexes.

**Table 4:** Goodness of fit of the invariance models

	χ2/df	RMSEA	SRMR	CFI
M1: Unrestricted Model	2.560	0.050	0.040	0.967
M2: Measurements Weight Model	2.636	0.051	0.050	0.963
M3: Measurement Intercepts Model	2.509	0.049	0.051	0.962
M4: Measurement Residuals Model	2.475	0.047	0.083	0.960

**Table 5:** Comparisons between invariance models

Comparison	|Δ RMSEA|	|Δ SRMR|	|Δ CFI|
M2 vs. M1	0.001	0.010	0.004
M3 vs. M2	0.002	0.001	0.001
M4 vs. M3	0.002	0.032	0.002

Note: Comparison criteria: ΔCFI < 0.005, ΔRMSEA < 0.010, ΔSRMR < 0.025

### Sociodemographic predictors and behavioural outcomes of the HEAS-PT

The correlation matrix of the study variables is presented in Additional File 2, illustrating the bivariate correlations between variables. Hochberg correction for multiple comparisons established the α level at 0.001.

Sex, age and living area were not significantly related to the HEAS-PT subscales. Even though schooling did not relate to any subscale, paternal education attainment was positively related to personal impact.

Multiple linear regressions in which all sociodemographic variables (gender, age, schooling, living area, fathers’ school attainment and mothers’ school attainment) were entered were further performed to statistically predict each subscale. Bonferroni correction for multiple comparisons established the α level at 0.008. The affective symptoms, rumination and behavioural symptoms subscale models were not significantly explained by any predictors. The only subscale that was significantly explained was personal impact, which was predicted by the paternal education attainment, providing further evidence for the previous bivariate analysis (*R*^2^ = 0.026, *p* = .012, *B* = 0.878, *p* = .001).

Bivariate correlation analyses showed that higher scores on all subscales of the HEAS-PT were moderately related to pro-environmental behaviours. A multiple linear regression to test the unique effects of the HEAS-PT subscales was conducted (Table 6). Multicollinearity between subscales could be a problem; therefore, it was assessed by examining tolerance and the variance inflation factor (VIF) [38]. The tolerance value was above 0.1 (0.441–0.599), and the VIF was below 5 (1.668–2.268), suggesting that there was no multicollinearity. The analysis confirmed that rumination and anxiety regarding personal impact uniquely predicted pro-environmental behaviour (*R*^2^ = 0.119, *p* < .001).

**Table 6:** Multiple linear regression analysis: Predicting pro-environmental behaviour

	Estimate (B)	SE	95% CI	*p*
**Subscales**				
Intercept	1.149	0.046	(1.059–1.238)	< 0.001
Affective S.	-0.012	0.016	(-0.043–0.019)	0.455
Rumination	0.066	0.020	(0.027–0.105)	< 0.001
Behavioural S.	0.035	0.017	(0.001–0.070)	0.043
Personal impact	0.059	0.016	(0.028–0.090)	< 0.001

SE: Standard error. CI: Confidence interval. S.: symptoms. Bonferroni correction for multiple comparisons established the α level at 0.0125

## Discussion

This study presented a validated Portuguese version of the HEAS, the HEAS-PT, demonstrating favourable psychometric properties of this translated scale. The good fit of the observed data to the predicted model confirms that the HEAS-PT reproduces the scale’s original structure. Moreover, strong factor loadings showed that the variables correlate significantly with the factors underlying the construct. Good internal consistency values were observed, indicating that the various items included in the HEAS-PT dimensions captured the same construct with values similar to those of the original scale [7]. The intraclass correlation coefficients confirmed the reliability over time. Hogg and colleagues [7] found that affective and behavioural symptoms were less stable over time than rumination and anxiety regarding personal impact, as indicated by weaker ICCs [7]. In the present study, rumination and behavioural symptoms were less stable over time than affective symptoms and anxiety regarding personal impact. Anxiety regarding personal impact had the largest ICC values in both studies, suggesting that concern about one’s contribution to climate change is the most stable component of eco-anxiety over time. On the other hand, affective symptoms were the least stable factor over time in the original scale and the second most stable factor in our sample. One possible explanation is the greater time elapsed between measurement instances in the original study (12 weeks versus 7–10 days in our study). Thus, perhaps the fluctuation in eco-anxiety dimensions occurs along different time courses. Another possible explanation for this concerns the cultural differences between the samples, where the persistence of the Portuguese sample’s affective symptoms may derive from the recent publication of the Portuguese Climate Base Law [39]. Behavioural symptoms of eco-anxiety fluctuated more in our study and the original validation study [7], and further investigation of this dimension is warranted. We believe that the use of coping strategies by young adults in response to stressful stimuli or climatic/environmental variations they experience may explain the instability in their responses.

The scale’s results showed a strict invariance between men and women. Thus, the level of eco-anxiety in a sample may be generalizable between the two sexes. Therefore, the validated scale is an appropriate psychometric instrument to assess eco-anxiety in Portuguese youth. Further validation with older adults can establish whether young people experience greater eco-anxiety than older people and whether the experience is similar or dissimilar across age groups.

Individuals with more highly educated fathers experienced higher levels of eco-anxiety, particularly higher levels of anxiety related to personal impact. Therefore, it is crucial to explore the reasons for this relationship. The literature is inconsistent regarding the association between paternal education level and child mental health. For example, the study by Park and Fuhrer [40] clarified that there is no association between paternal education level and child mental health, while Torvik et al. [41] and Sheikh et al. [42] each present contrary associations. The former points to low paternal education levels as a predictor of mental health disorders in children, whereas the latter reveals that high education in fathers is associated with lower well-being in adulthood. However, eco-anxiety is not a clinical disorder or indicative of poor mental health.

The produced results suggest that fathers with high levels of education can instil greater attention to climate change in their children, and this heightened awareness may make them more likely to experience eco-anxiety. According to Meyer [43], people with higher levels of education tend to be more concerned about the environment and show more ecologically-conscious behaviours. In turn, several studies have shown a positive association between paternal education level and pro-environmental behaviours in children due to greater environmental knowledge [44–46].

The results showed that higher HEAS-PT scores were related to higher engagement in pro-environmental behaviours, consistent with other recent studies [47]. Although negative emotions can feel unpleasant [15], labelling eco-emotions as “psychoneurotic illnesses” [17, 48] could falsely imply that these experiences are inherently pathological. The severity and unresolved trajectory of environmental crises instead mean that feelings of concern and anxiety are appropriate and proportional to the threat [6, 7], and our results suggest that eco-anxiety can be seen as a response to protect the planet rather than a maladaptive response to climate change. Of the dimensions, rumination and anxiety about personal impact each predicted pro-environmental behaviour. Rumination is an important aspect of both eco-anxiety and climate change anxiety and shows that ecological problems are at the forefront of young people’s minds [6, 7]. Furthermore, rumination is strongly associated with anxious symptomatology [49]. According to Riley and colleagues [50], rumination can promote action and thus may represent an “impetus” for healthy people to act on behalf of the environment. However, for those who generally experience greater psychological distress, rumination may hinder behaviour, as thinking repetitively about ecological problems may exacerbate preexisting distress. Thus, exploring these relationships in clinical populations is warranted [51]. Furthermore, anxiety regarding personal impact predicted pro-environmental behaviours. Consistent with this finding, the perceived behavioural control component of the theory of planned behaviour is associated with the adoption of pro-environmental behaviour [52].

This study has limitations. First, the evaluation of the test-retest reliability of the HEAS-PT can be considered limited. Although the time interval used between tests is frequently reported in the literature, it is shorter than the most commonly recommended test-retest interval of two weeks between measurement points [53]. Usually, the smaller the time interval between tests, the greater the test-retest reliability. In short periods of time, there is a risk of temporal stability in respondents’ answers due to their memory of the questions and answers given in the initial test. Nonetheless, the HEAS assesses eco-anxiety felt over the previous two weeks; thus, considering some research points out anxiety symptoms as an unstable construct [54], and there is no research to our knowledge regarding the stability of eco-anxiety, we opted to slightly shortening the interval between tests. Moreover, our correlational analyses of the likely demographic antecedents and pro-environmental consequences of eco-anxiety were cross-sectional. A longitudinal evaluation of these variables would capture change within subjects over time and thus give some advantage to causal modelling in terms of providing more knowledge to establish causal relationships [55]. That would provide more robust scientific evidence on the potential causes and consequences of eco-anxiety.

Even though the convenience sampling technique did not guarantee the representativeness of the sample, we used a convenience sample of a homogeneous population (i.e., a sample that was intentionally limited to a specific sociodemographic subgroup – aged between 18 and 25 years) which, according to some researchers, is more likely to be representative than convenience samples of heterogeneous populations [56]. Regarding the potential lack of representativeness of the sample, for example, the number of young adults engaged in environmental associations was small (n = 6), hindering a comparison between eco-anxiety in people who were and were not engaged in environmental associations. In these two groups, there could be significant differences in the levels of eco-anxiety because of different levels of involvement in environmental issues. Thus, future studies should intentionally recruit members of environmental organisations to enable comparability. Understanding the vulnerability and protective factors of individuals proactively addressing the world’s ecological challenges would contribute to supporting environmental activists in their endeavours. Finally, our study focused on young adults, so our findings may not – and were never intended to – generalise to other age groups. A synthesis of the extant literature supports the psychometric properties of the HEAS across wider ranges of ages than we included here [11]. Younger generations tend to report higher levels of eco-anxiety [57], and thus we prioritised developing a tool for use with Portuguese youth in the first instance.

Although there are anxiety assessment instruments for the Portuguese population with good psychometric properties, it was not possible to identify any tool that specifically measures eco-anxiety. Thus, the HEAS-PT can be effectively used to represent this phenomenon. Mild levels of eco-anxiety can be seen as a normal and adaptive response to climate change, which may prompt protection of the environment. However, if debilitating levels of eco-anxiety are experienced, people may need support to build resilience, act, make social connections, receive emotional support, and connect with nature [58]. Consistent evidence about this emotional problem may also provide further justification for urgent and impactful political decisions related to environmental and health matters aimed at protecting people and the planet.

## Conclusions

This paper highlights a measurement instrument of a phenomenon of global interest: eco-anxiety. The psychometric properties of the HEAS-PT were evaluated, and the scale was shown to be valid, reliable, and suitable for use with young Portuguese adults. We encourage the use of the HEAS-PT in future research and direct interested readers to Additional File 1 for a copy of the original English version of the scale and our newly validated European Portuguese translation.

In addition, paternal education level also predicted eco-anxiety, particularly anxiety about one’s personal environmental impact. Therefore, education seems vital to environmental sustainability, as it can raise awareness of environmental issues. Importantly, eco-anxiety predicted pro-environmental behaviours. Thus, concerns about climate change should be viewed as adaptive and protective responses of individuals to planetary protection.

### Electronic supplementary material

Below is the link to the electronic supplementary material.

Supplementary Material 1

Supplementary Material 2

## Data Availability

The datasets used and/or analysed during the current study are available from the corresponding author upon reasonable request.
