# Supplementary material for: Validating a measure for eco-anxiety in Portuguese young adults and exploring its associations with environmental action
Source: BMC Public Health. 2023 Oct 2;23:1905. doi: 10.1186/s12889-023-16816-z (PMC10546781; doi:10.1186/s12889-023-16816-z)
Supplement: Supplementary file 1 — Supplementary Material 1 [file 12889_2023_16816_MOESM1_ESM.docx]

| **Semantic equivalence of the scale items in the original and translated versions** | | |
| --- | --- | --- |
|  | **English** | **Portuguese** |
| **Factor 1** | **Affective symptoms** | **Sintomas afetivos** |
| **Item 1** | Feeling nervous, anxious or on edge | Sentir-se nervoso(a), ansioso(a) ou no limite |
| **Item 2** | Not being able to stop or control worrying | Não ser capaz de parar ou controlar a preocupação |
| **Item 3** | Worrying too much | Preocupar-se em demasia |
| **Item 4** | Feeling afraid | Sentir-se com medo |
| **Factor 2** | **Rumination** | **Ruminação** |
| **Item 5** | Unable to stop thinking about future climate change and other global environmental problems | Ser incapaz de parar de pensar sobre alterações climáticas futuras e outros problemas ambientais globais |
| **Item 6** | Unable to stop thinking about past events related to climate change | Ser incapaz de parar de pensar sobre acontecimentos passados relacionados com as alterações climáticas |
| **Item 7** | Unable to stop thinking about losses to the environment | Ser incapaz de parar de pensar sobre perdas ambientais |
| **Factor 3** | **Behavioural symptoms** | **Sintomas comportamentais** |
| **Item 8** | Difficulty sleeping | Ter dificuldade em dormir |
| **Item 9** | Difficulty enjoying social situations with family and friends | Ter dificuldade em desfrutar de eventos sociais com a família e os amigos |
| **Item 10** | Difficulty working and/or studying | Ter dificuldade em trabalhar e/ou estudar |
| **Factor 4** | **Anxiety about personal impact** | **Ansiedade relacionada com o impacto pessoal** |
| **Item 11** | Feeling anxious about the impact of your personal behaviours on the earth | Sentir-se ansioso(a) relativamente ao impacto dos seus comportamentos individuais no planeta |
| **Item 12** | Feeling anxious about your personal responsibility to help address environmental problems | Sentir-se ansioso(a) relativamente à sua responsabilidade individual para ajudar a dar resposta aos problemas ambientais |
| **Item 13** | Feeling anxious that your personal behaviours will do little to help fix the problem | Sentir-se ansioso(a) pelos seus comportamentos individuais irem fazer pouco para ajudar a resolver o problema |
