# Supplementary material for: Validating a measure for eco-anxiety in Portuguese young adults and exploring its associations with environmental action
Source: BMC Public Health. 2023 Oct 2;23:1905. doi: 10.1186/s12889-023-16816-z (PMC10546781; doi:10.1186/s12889-023-16816-z)
Supplement: Supplementary file 2 — Supplementary Material 2 [file 12889_2023_16816_MOESM2_ESM.docx]

**Mean values or frequencies and correlations between variables (Pearson and Point biserial)**

| Variables | Mean / % |  | 1 | 2 | 3 | 4 | 5 | 6 | 7 | 8 | 9 | 10 | 11 |
| --- | --- | --- | --- | --- | --- | --- | --- | --- | --- | --- | --- | --- | --- |
|  |  |  |  |  |  |  |  |  |  |  |  |  |  |
| 1. Affective S. | 2.86 |  | - |  |  |  |  |  |  |  |  |  |  |
| 2. Rumination | 1.52 |  | .599*** | - |  |  |  |  |  |  |  |  |  |
| 3. Behavioural S. | 1.60 |  | .646*** | .467*** | - |  |  |  |  |  |  |  |  |
| 4. Personal impact | 2.65 |  | .530*** | .593*** | .360*** | - |  |  |  |  |  |  |  |
| 5. Sex ^a^ | 81.5% |  | -.070 | -.009 | -.032 | .006 | - |  |  |  |  |  |  |
| 6. Age | 20.46 |  | -.064 | -.005 | -.084 | .023 | .062 | - |  |  |  |  |  |
| 7. Education ^b^ | 13.24 |  | -.003 | .002 | -.064 | -.008 | .036 | .519 | - |  |  |  |  |
| 8. Residence area ^c^ | 28.7% |  | -.060 | -.045 | -.072 | -.023 | .074 | -.004 | .005 | - |  |  |  |
| 9. Paternal’ EL ^d^ | 80.4% |  | .083 | .078 | .103 | .151*** | .036 | .029 | .064 | .143*** | - |  |  |
| 10. Maternal’ EL ^d^ | 70.5% |  | .059 | .067 | .071 | .083 | .000 | .019 | .018 | .162*** | .505*** | - |  |
| 11. PEB | 1.46 |  | .223*** | .302*** | .220*** | .299*** | -.77 | -.065 | -.008 | -.056 | .020 | 0.15 | - |

EL: education level. PEB = Pro-environmental behaviour. ^a^ 0 = female, 1 = male; % of female is presented. ^b^ Number of Years. ^c^ 0 = rural, 1 = urban; % of rural is presented ^d^ 0 = until secondary level, 1 = tertiary level; % of secondary level is presented. Using Hochberg correction to account for multiple analysis, the α level was set at .001.

*** *p* < 0.001
